# Congruent morphological and genetic differentiation as a signature of range expansion in a fragmented landscape

**DOI:** 10.1002/ece3.787

**Published:** 2013-09-25

**Authors:** Ronan Ledevin, Virginie Millien

**Affiliations:** Redpath Museum, McGill University859 Sherbrooke Street West, Montreal, H3A 0C4, QC, Canada

**Keywords:** Climate change, morphometrics, range shift, white-footed mouse

## Abstract

Phenotypic differentiation is often interpreted as a result of local adaptation of individuals to their environment. Here, we investigated the skull morphological differentiation in 11 populations of the white-footed mouse (*Peromyscus leucopus*). These populations were sampled in an agricultural landscape in the Montérégie region (Québec, Canada), at the northern edge of the distribution of the white-footed mouse. We found a strong pattern of phenotypic differentiation matching the genetic structure across these populations. Landscape fragmentation and the presence of geographic barriers, in particular north–south oriented rivers, contribute to this differentiation and modulate the pattern of rapid ongoing northward range expansion of the white-footed mouse in response to climate warming. We conclude that while large rivers and postglacial recolonization routes have shaped the current pattern of distribution and differentiation of white-footed mouse populations, further local differentiation is occurring, at the scale of the landscape. We posit that the northern expansion of the white-footed mouse is achieved through successive independent founder events in a fragmented landscape at the northern range edge of the species. The phenotypic differentiation we observe is thus a result of a number of mechanisms operating at different spatial and temporal scales.

## Introduction

Patterns of geographic variation in morphological and genetic diversity of a species reflect both past history and recent evolutionary processes. In a context of environmental change, organisms can either adapt locally, migrate, or become extinct (Davis et al. 2005). In the past decades, the ability of organisms to respond to drastic and rapid climate change has been challenged (e.g., Hughes et al. 2003; Ryan and Cunningham 2012; Vavrus et al. 2012). Overall, models predict a global increase of up to 4°C by the end of the XXIst century (New et al. 2011). However, if an increase in average temperature can have dramatic impacts on cold-adapted species (Derocher et al. 2013), it can also favor the expansion of temperate ones (Parmesan 2006; Berteaux et al. 2010).

The effects of environmental changes on organisms can be detected at different temporal scales (Millien et al. 2006; Teplitsky and Millien In press). The influence of long-term climatic trends has been investigated in a number of phylogeographic studies, an approach helpful at identifying glacial refugia and postglacial recolonization routes (e.g., Avise et al. 1987; Taberlet et al. 1998). For example, we have a good understanding of the impact of the last glaciation, about 20,000 years ago, on many European and North American species in these regions (e.g., Deffontaine et al. 2005; Koblmüller et al. 2012). There is an increasing number of studies in which morphological and genetic variation are integrated (e.g., DeLeon et al. 2012). Congruent patterns of variation at the phenotypic and molecular levels have been reported, both reflecting postglacial history of the species (e.g., Gündüz et al. 2007; Deffontaine et al. 2009; Ledevin et al. 2010). While distinct patterns of phenotypic and molecular variability hint toward the effect of selection on the phenotypic trait studied, such consistent patterns can result from either selection or stochastic events (Xu et al. 2010; Teplitsky and Millien In press). An integrative approach combining molecular and morphological data can thus help better identifying and understanding the mechanisms shaping species differentiation.

In the current context of rapid environmental change coupled with increasing landscape modifications due to human activities, it has become critical to better estimate the ability of species to respond to these changes on a very short-time scale. There is growing evidence that rapid morphological changes are expected as a response to recent global change (Berteaux et al. 2004; Bradshaw and Holzapfel 2006; Sheridan and Bickford 2011). For example, rapid morphological changes correlated with climatic changes over several years were reported for two heteromyid rodents (Wolf et al. 2009), Soya sheep (Ozgul et al. 2009), yellow-bellied marmots (Ozgul et al. 2010), or barn swallow (Møller and Szép 2005).

To investigate the influence of long- and short-term climatic changes on morphology, we used the white-footed mouse (*Peromyscus leucopus* Rafinesque, 1818) as a model and quantified the skull shape of specimens trapped in southern Québec, Canada. *Peromyscus leucopus* is a temperate generalist rodent found in diverse habitats, with a preference for forests (Desrosiers et al. 2002). The white-footed mouse population structure is affected by landscape modifications and habitat fragmentation in southern Québec (Rogic et al. 2013; R. R. Marrotte, A. Gonzalez, and V. Millien, unpubl. data). In addition, its distribution has expanded northward in the region, at a rate estimated at 10 km per year (E. Roy-Dufresne, L. Travis, J. A. Simon, G. L. Chmura, and V. Millien, unpubl. data). Our sampling sites are also located at a crossroad between different postglacial recolonization pathways for the species, out of at least two distinct refugia (Rowe et al. 2006; J. Fiset, N. Tessier, V. Millien, and F. -J. Lapointe, unpubl. data). The study of white-footed mice populations in southern Québec therefore provides an excellent opportunity to evaluate the relative and combined effects of recent and past climate change combined with alterations of the landscape on species phenotypic differentiation.

We performed a morphometric analysis of the skull of *P. leucopus* individuals from 11 populations in a fragmented landscape to assess the pattern of morphological differentiation across this landscape and compare the genetic structure of these populations with this pattern of morphological differentiation. We first investigated the role played by natural (i.e., rivers) and anthropogenic (i.e., roads) landscape features that have been shown to be efficient barriers to dispersal for the white-footed mouse in our study area (Rogic et al. 2013; R. R. Marrotte, A. Gonzalez, and V. Millien, unpubl. data). We then discuss whether the observed pattern of phenotypic variation has been shaped by short-term environmental changes (anthropic effect), historical processes (the recent northern expansion of the mouse), long-term changes (Quaternary glaciations), or a combination of them. More specifically, we addressed the hypotheses that: (1) there is a congruent pattern of differentiation among the white-footed mouse populations at the genetic and phenotypic levels; (2) landscape fragmentation (i.e., presence of rivers, roads or agricultural matrix), recent northern range shift of *P. leucopus* and its postglacial pattern of recolonization all contributed to the observed pattern of morphological differentiation; and (3) local habitat fragmentation is preventing the occurrence of a continuous front of colonization, and the northern expansion of the white-footed mouse is achieved by successive independent founding events.

## Materials and Methods

### Specimens and study sites

**Figure 1:**
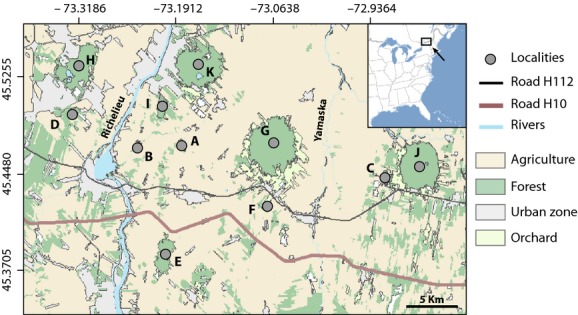
Sampling localities and main landscape characteristics in the study area. The Monteregian Hills are Mont Saint Bruno (H), Mont Saint Hilaire (K), Mont Rougemont (G), and Mont Yamaska (J). Groups of localities were defined from the genetic structure of the populations from Rogic et al. (2013) as follow: central (A, B, G, I, K), south (E, F), west (D, H) and east (C, J).

We used a total of 332 specimens of white-footed mouse (*P. leucopus*), trapped between 2007 and 2011 at 11 localities from the Montérégie region, southern Québec (Fig. 1, Table 1). The study area covers 634 km^2^, and among the 11 localities, four are part of the Monteregian Hills while other localities are forest fragments around these hills. Only adult specimens with the third molar erupted were considered.

**Table 1:** Sampling localities with the total number of *Peromyscus leucopus* as well as the number of males and females at each site. The group variable was defined from the genetic structure of the populations from Rogic et al. (2013)

Site	Group	*M*	*F*	Ntot.
A	Center	23	10	33
B	Center	16	14	30
C	East	19	14	33
D	West	13	15	28
E	South	15	17	32
F	South	19	14	33
G	Center	27	5	32
H	West	24	9	33
I	Center	17	14	31
J	East	10	4	14
K	Center	28	5	33

The population genetic structure of these populations was previously described using 11 microsatellites *loci* (Rogic et al. 2013). The same localities and specimens were used here to quantify the morphological differentiation, depending on the state of preservation of the skulls (broken skulls were removed from the morphological dataset), allowing the comparison of phenotypic and genetic patterns of variation across these populations. Several geographic barriers to gene flow were identified in the study area (Rogic et al. 2013; Marrotte R. R., Gonzalez, A. and Millien, V., unpubl. data), that may promote morphological differentiation across populations. Here, we focus on the effect of two rivers (the Richelieu and Yamaska rivers) and two roads (H112 and H10).

### Shape analysis

**Figure 2:**
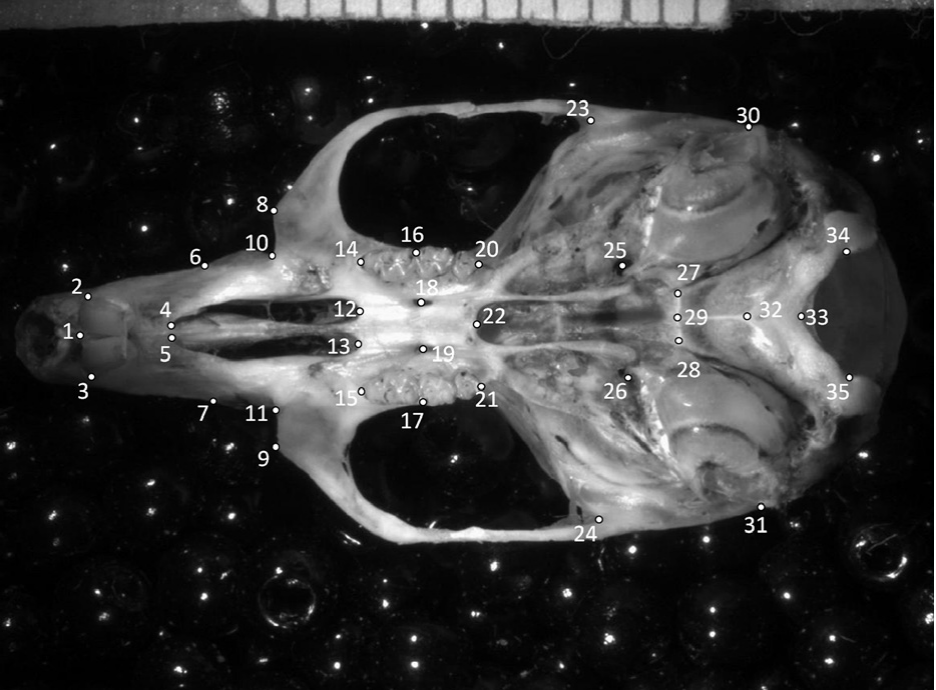
Landmark configuration on the ventral side of the white-footed mouse's skull. (1) Mid-point on premaxilla, between anterior part of the incisors; (2–3) Upper lateral extremity of the incisor alveolus; (4–5) Anterior extremity of the incisive foramen; (6–7) Lateral extremity of the premaxilla-maxilla suture; (8–9) Angle inflexion on the maxillary arm; (10–11) Insertion of the maxillary arm on the rostrum; (12–13) Posterior extremity of the incisive foramen; (14–15) Anterior extremity of the dental tooth row; (16–17) Labial-side point between first and second upper molars; (18–19) Posterior palatine foramen; (20–21) Posterior extremity of the dental tooth row; (22) Median point on the posterior margin of the palate; (23–24) Anterior maximum of curvature of the squamosal; (25–26) Foramen ovale; (27–28) Meeting point between the basisphenoid, basioccipital and tympanic bulla; (29) Mid-point of the basioccipital–basisphenoid suture; (30–31) Posterior tip of the external auditory meatus; (32) Mid-basioccipital point; (33) Basion; (34–35) Internal flexion of the occipital condyle.

A set of 35 landmarks was used to describe the ventral view of the cranium (Fig. 2). All specimens were measured by the same operator (RL). The original coordinates were standardized for size, positioning in space and skull orientation using a generalized Procrustes analysis (Rohlf and Slice 1990). Size information was retained as centroid size (CS), the square root of the sum of squared distances between each landmark and the centroid of the landmark configuration. We used the thin plate spline algorithm (TPS: Bookstein 1991) to visualize shape differences, and computed deformation grids using the least bending energy criterion between the consensus and target landmark configurations.

### Statistical analyses

We investigated size differences using univariate statistics. No sexual dimorphism in size or significant relation between size and environmental variables was detected; thus, only shape was considered in further analyses.

Multivariate statistics were used to investigate shape differentiation of the skull. We first tested for the influence of allometry in our data using a multivariate regression between the CS and the shape variables. We found a significant allometric effect (*P* < 0.001) and thus used the residuals of the regression as a new set of “allometry-free” variables. We first tested for the effect of sexual dimorphism on shape with a multivariate analysis of variance (MANOVA). We then performed a between-group Principal Component Analysis (PCA) on the residuals of the regression (gpPCA [Boulesteix 2004; Mitteroecker and Bookstein 2011]), using the major geographic groups identified genetically as a grouping variable (Table 1). The gpPCA provides an alternative to the linear discriminant analysis (LDA), a method widely questioned in morphometrics (e.g., Klingenberg & Monteiro, 2005; Mitteroecker and Bookstein 2011). The Procrustes geometry is not preserved in canonical variate analyses (CVA) ordinations used to estimate the discriminant functions. Instead, the gpPCA, using projection of the data onto the principal components of the group means, keeps the axes orthogonal and thus allows interpretations of shape variation along the axes in a biologically meaningful way. A MANOVA was performed to evaluate the overall intergroup differentiation, and permutations tests were used to test for pairwise group divergence.

A matrix of pairwise distances was then calculated from the shape coordinates and compared with the matrix of genetic distances (*F*_st_) from Rogic et al. (2013), using a Mantel test with 1000 permutations (Mantel 1967). Phenetic relationships were investigated through cluster analysis performed on the matrix of Mahalanobis distances using an UPGMA method and the nearest neighbor clustering algorithm in the *cluster* package in R (R Development Core Team 2013).

We carried out a multiple regression on distance matrices (MRM as described in Legendre and Legendre 1998; Lichstein 2006) using the *ecodist* package in R (R Development Core Team 2013) to evaluate the effect of rivers and roads on the morphological structure. Series of dummy variables [0, 1] were used to characterize whether sampling localities were on one side or the other of each barrier. Distance matrices were then computed from these data and compared with the matrix of morphological Mahalanobis distances.

## Results

### Sexual dimorphism

No sexual dimorphism was detected for skull shape, and the interaction between sex and localities was not significant (Table 2). Males and females were thus pooled together in further analyses.

**Table 2:** Multivariate analysis of variance analysis testing for the effect of sexual dimorphism and sampling site on the skull shape

	Wilks' λ	Pr(>*F*)
Sex	0.917	0.166
Locality	0.252	<2e-16
Sex^*^locality	0.493	0.224

### Interpopulations shape differentiation

**Figure 3:**
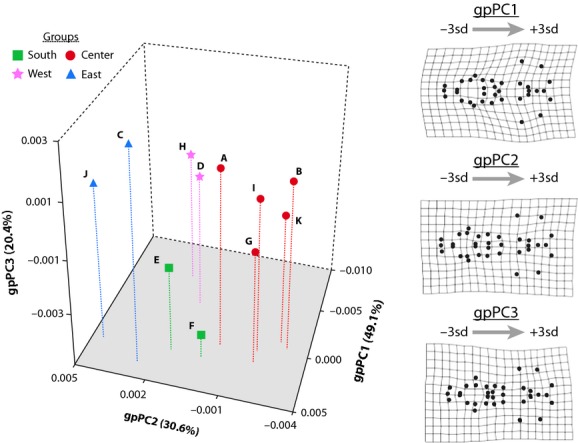
Scatterplot of the between-groups principal component analysis. The first three axes (gpPCs) and the percentage of variance they explain are indicated. Each symbol corresponds to the mean by locality, and the groups are coded by color. On the right side of the figure, the shape changes occurring along the three axes are represented using thin plate spline deformation grids. The grids represent the shape changes along each axis (from the most negative to the most positive configuration ± 3 standard deviation to magnify the differences).

A gpPCA performed on the allometry-free shape variables with the four clusters identified in Rogic et al. (2013) as groups (Table 1) revealed that the skull morphology differed significantly between these groups (Wilks' λ = 0.338, *P* < 0.001). The most divergent populations were sites D and H (Table 3), which were located on the western side of the Richelieu River and differentiated along the first axis gpPC1 (49.1% of total variance). The skull shape reconstructions showed an elongation of the rostrum and a broadening of the neurocranium along this axis (Fig. 3).

**Table 3:** Matrix of *P*-values for pairwise comparisons between groups using permutation tests on the Euclidian distances between group means

	Central	East	South
East	0.002		
South	0.020	0.038	
West	0.001	0.001	0.001

The second axis gpPC2 (30.6% of total variance) mostly reflected the differentiation between the eastern (positive values) and central (negative values) groups (Fig. 3), these two groups being significantly different (Table 3). Southern localities (sites E and F) were intermediate on gpPC2 axis, between the eastern and central ones. The deformation grids revealed small and localized changes along this axis, mostly around the palatal area, which is more extended in eastern populations (Fig. 3).

The southern group differentiated from other groups along the third axis gpPC3 (20.3% of total variance), although the differentiation along this axis was the least significant (Table 3). A narrowing of the neurocranium and a broadening of the rostrum was observed from negative to positive values along this axis (Fig. 3).

We compared the matrix of shape distances based on Mahalanobis distances calculated from the shape coordinates with a matrix of geographic distances and found a significant pattern of isolation by distance among the 11 populations (Mantel *r* = 0.594, *P* < 0.001). We then performed a MRM and found a significant pattern of isolation by barrier in our data (*R*^2^ = 0.566, *P* = 0.009). The largest river (the Richelieu) had the strongest effect on the morphological differentiation, followed by the Yamaska River (Table 4). We did not detect any significant effect of the roads H112 and H10 with this analysis, although permutation tests indicated a differentiation of populations south of H112 from all other populations (Table 3).

**Table 4:** Results of the multiple regression on distance matrices performed on morphological distances with the barrier matrices as explanatory variables

Barrier	Estimate	*P* value
Richelieu river	1.21E-05	0.0001
Yamaska river	3.30E-05	0.0085
Highway 10	−1.40E-05	0.2003
Highway 112	1.85E-05	0.0839

### Morphology versus genetic differentiation

We compared the average shape differentiation of the populations with *F*_st_ values from Rogic et al. (2013) to evaluate the congruence of the pairwise morphological and genetic differentiation between our populations. The phenotypic and genetic distance matrices were significantly correlated (Mantel *r* = 0.39, *P* = 0.01).

**Figure 4:**
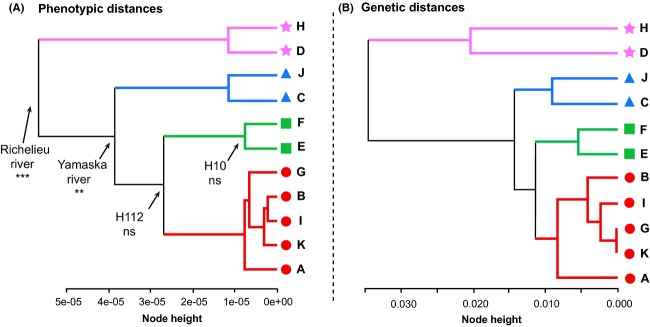
Hierarchical clustering phenograms computed on (A) the matrix of phenotypic distances (nearest neighbor), and (B) the matrix of genetic distances (UPGMA), modified after Rogic et al. (2013). The significance level obtained in the multiple regression on distance matrices for each geographical barrier is given for the morphological data at each node on the tree.

A cluster analysis was then performed to compare the patterns of interpopulations differentiation based on Mahalanobis shape distances and *F*_st_ values (Fig. 4), and we obtained very similar topologies. In both phenotypic and genetic trees, the western localities branched deep in the tree, confirming the key role played by the Richelieu River as a geographic barrier enhancing differentiation. The second diverging group was the eastern one, isolated from the other groups by the Yamaska River. The southern and central groups were the most closely related, presenting low phenotypic and genetic differentiation.

## Discussion

Despite the small geographic extent of our study, we detected a significant morphological structure across Monteregie populations, matching the genetic differentiation observed in these populations (Rogic et al. 2013). Most morphological differentiation was through an elongation of the rostrum and a broadening of the skull in the two populations west of the Richelieu River. Further changes in the palatal area differentiated the populations east of the Yamaska River from populations south of Highway 10 and the central populations. Such changes in the relative length of the rostrum and the palatal area or changes in the width of the braincase are likely related with feeding or sensory functions. However, we cannot establish that selection is actually driving the morphological differentiation in our white-footed mouse populations, and no further conclusions can be drawn on the functional meaning of the morphological changes we observed. Yet, it remains that populations that were similar in their genetic composition were also more similar in the shape of the skull. Such high congruence between phenotypic traits and microsatellites has not often been reported. When discrepancies between the two were observed, the phenotypic divergence was usually attributed to variations in the local environment or food availability (Lalis et al. 2009; DeLeon et al. 2012; Sistrom et al. 2012). Divergent patterns of phenotypic and genotypic differentiation suggest that the phenotypic trait studied is under selection. Alternatively, similar patterns of molecular and phenotypic differentiation have been related both to random genetic drift or natural selection experienced by geographically separated populations (Smith et al. 2005; Milá et al. 2009; Ortego et al. 2011). The comparison of *Q*_st_ and *F*_st_ values could be used to detect the effect of selection on individuals (but see Whitlock 2008), but it requires a study design (i.e., common garden experimental setting) that is not readily available when studying historical patterns of differentiation from museum specimens. Instead, an approach integrating several distinct levels of differentiation, both morphological and molecular, can hint toward the mechanism driving differentiation in the study samples. Here, we suggest that local differentiation is the result of successive independent founder events (Bell 2013), a mechanism especially relevant in a context of range expansion. The congruence observed in *P. leucopus* between nuclear markers and the phenotype is likely related with habitat fragmentation in the region (as seen in Dujardin 2008), as well as larger scale responses to environmental change.

### Effect of geographic barriers

Geographic barriers such as the Richelieu and the Yamaska rivers play a major role in the population spatial differentiation. The white-footed mouse is able to swim, but not very efficiently (Klee et al. 2004), and there is no evidence that large rivers can be crossed by mice. Following the Horton–Strahler stream ordering (Horton 1945; Strahler 1952; Peckham and Gupta 1999), the Richelieu River is estimated to be of fifth order or higher, and the Yamaska River to be at least of fourth order. Although smaller than the Richelieu, the Yamaska River is wide enough to constitute a significant barrier to dispersal for the mouse. The differentiations we observed between the western and central groups and between the eastern and central groups are hence interpreted as the result of these two strong geographic barriers oriented south–north.

Frequently evidenced as efficient barriers, especially in low-mobility species such as amphibians (Baur and Baur 1989), roads have also proven to affect bigger animals such as the Eurasian lynx (Kramer-Schadt et al. 2004), bobcats or coyotes (Riley et al. 2006). Contrary to rivers, roads did not appear to be as strong barriers to dispersal for *P. leucopus*. Southern populations diverged in their skull morphology from other groups, but this difference was not large. Such a small barrier effect was also found for highway H112 using microsatellites (Rogic et al. 2013). Supporting these results, (McGregor et al. 2007) evidenced that small mammals tends to avoid roads, although some individuals are able to cross highways. Similarly, in our study system, roads thus tend to impede dispersal in *P. leucopus*, but not as strongly as rivers.

### Effect of the connectivity of the habitat

We found that southern populations were intermediate in their morphology between Eastern and central ones. This pattern can be explained by the presence of numerous forest patches south of our study area, creating an effective southern connection between the eastern and western groups, through the southern localities. If the white-footed mouse is found mostly in forests, it is also frequently observed in diverse habitats such as open fields, grass, road ditches, corn fields, or human habitations (Cummings and Vessey 1994). This diversity of habitat used by the mouse increases its dispersal ability, and individuals are able to disperse over a wide range of distances throughout their life span, between 85 and 867 m (Krohne and Hoch 1999). Forest fragments in our sampling area tend to connect in the south, and most of the interpatches distances are lower than 900 m. The mouse is thus likely able to use the multiple forest patches as stepping stones as a mean to disperse over large distances, a very efficient colonization process too often neglected (Baum et al. 2004).

### Effect of the habitat in the landscape

A vast majority (63%) of our study extent is covered by agricultural fields, and our sampling sites thus represent woodland areas within a “sea of agriculture”. Intense agricultural practices began in the 1940s and led to a 70% decrease in the local forests in the region (Wampach 1988). Open fields negatively affect *P. leucopus* movement (Rizkalla and Swihart 2007), which is expected to enhance population differentiation. Most mouse individuals prefer to use corridors and are unlikely to willingly enter the agricultural matrix (Rizkalla and Swihart 2007). However, the genetic study performed on our samples (Rogic et al. 2013) did not evidence that white-footed mice in isolated woodlots suffered decreased genetic variation, as it would be expected if dispersal was substantially inhibited by the agricultural matrix (Mossman and Waser 2001). Similarly, we found a strong phenotypic similarity between central populations that are all located in an agriculture-dominated landscape. The influence of an agricultural landscape on mice dispersal appears thus to be limited, which may be in part due to the existence of seasonal corridors within the landscape. Depending on crop height and maturity, they can act as dispersal barrier (Krohne and Hoch 1999) or corridors (Cummings and Vessey 1994; Anderson et al. 2003), explaining the low genetic and morphological differentiation between the populations sampled in forest patches within an agricultural landscape.

### Recent range expansion

Recent climate change deeply affect species geographic distribution, and there is strong empirical evidence for range expansion in Northern Hemisphere temperate species driven by climate modifications (e.g., Thomas and Lennon 1999; Hill et al. 2002; Parmesan 2006). Although relatively rare in the past (Grant 1976), the white-footed mouse has become more abundant in the region over the last few decades (Rogic et al. 2013). The northern range limit of this species has been shifting north recently (Myers et al. 2009), and the northward expansion of *P. leucopus* is estimated to occur at a rate of 10 km per year in southern Quebec (E. Roy-Dufresne, L. Travis, J. A. Simon, G. L. Chmura, and V. Millien, unpublished data). The correlation between morphological and genetic patterns of differentiation we observed among *P. leucopus* populations may thus reflect the ongoing expansion of the white-footed mouse *via* small founder populations, independently colonizing different forest patches. Our finding of a significant isolation by distance further supports this hypothesis. In other words, we posit that the fragmentation of the landscape and the local habitat modulate the pattern of northward colonization observed in *P. leucopus*. Instead of a homogeneous front of migration, our results point to the existence of many propagules, small founding populations colonizing one forest patch after another. Further analyses have to be performed to test whether such a mechanism promoting local differentiation of populations exists in our study system, but the congruence between nuclear neutral markers and phenotypic traits supports this hypothesis. Coupling morphological data to genetic analysis could be an efficient way to study the process of range expansion, mostly addressed genetically so far (e.g., Edmonds et al. 2004; Excoffier and Ray 2008; Hallatschek and Nelson 2008; Peter and Slatkin 2013).

### The white-footed mouse postglacial history

In addition to recent climate warming, larger scale factors may also play an important role in the distribution and differentiation of *P. leucopus* at the most northern part of its range. Global warming since the last glacial maximum, about 20,000 years ago, has influenced species distribution (Taberlet et al. 1998; Hewitt 2004). Similarly, the postglacial history of the white-footed mouse has shaped its current pattern of distribution and most likely its morphological differentiation. Here, we found that the Richelieu River currently acts as a strong geographic barrier for the mouse. The morphological differentiation of populations located on each side of the river could have occurred recently through isolation by the river. However, the postglacial recolonization patterns of the white-footed mouse may also have resulted in such a geographic differentiation. Based on mitochondrial DNA, two clades of the white-footed mouse are present in our study area, along the northern and southern shores of the St-Lawrence River, respectively (J. Fiset, N. Tessier, V. Millien, and F. -J. Lapointe, unpubl. data). These two lineages are related to the central and north-eastern North American clades evidenced in Rowe et al. (2006), showing that the *P. leucopus* populations currently in Quebec originated from two distinct glacial refugia. Following the Horton–Strahler stream ordering (Horton 1945; Peckham and Gupta 1999), the Saint Lawrence River is estimated to be a 10th order river and is very unlikely to be crossed by mice. The Richelieu is only a fifth order river, but also constitutes a strong barrier to dispersal for the mouse. In sum, the current pattern of genetic and morphological differentiation of the white-footed mouse in southern Quebec is likely the result of the pattern of postglacial recolonization from distinct southern refugia, modulated by the presence of large north–south oriented rivers.

## Conclusions

The pattern of strong interpopulation morphological differentiation we found here was unexpected at such a small geographic scale. We conclude that while large rivers and postglacial recolonization routes have shaped the current distribution and differentiation of *P. leucopus* populations, further local differentiation is occurring, at the scale of the landscape. The fragmentation of the landscape, in particular the presence of north–south oriented rivers, contributes to this differentiation and is modulating the northward shift in the geographic range of *P. leucopus* in response to the global warming (Myers et al. 2009). Landscape fragmentation at the northern range edge of the species prevents the occurrence of a continuous front of colonization and northern expansion is thus achieved through successive independent founding events. This observation is of importance if we are to implement faunal pathways as a mitigation measure in response to global change (e.g., Nuñez et al. 2013), and such south–north corridors may allow a better preservation of genetic and phenotypic diversity during the process of expansion. Furthermore, morphological variation is often interpreted as an adaptive response to the environment, such as specific food, habitat resources, or the climate (e.g., Renaud and Millien 2001; Millien and Damuth 2004; Millien 2004; Pergams and Lawler 2009; Ravinet et al. 2012; Renaud et al. 2013). However, the morphological differentiation observed across populations at the northern limit of the species range is not necessarily a signature of local adaptation. In the context of range expansion, the hypothesis of multiple independent founder events at the front of invasion with no adaptive component cannot be ruled out. Finally, both range expansion and local adaptation could play in concert and produce a geographic structure, such as the one we observed here in the white-footed mouse. The integration of phenotypic, mtDNA, and nDNA data also pointed to the overlooked signature of postglacial recolonization in shaping the current patterns of species geographic differentiation. It is likely that the pattern of postglacial recolonization from distinct glacial refugia we described for the white-footed mouse also occurred in many other North American species (e.g., Zamudio and Savage 2003), thus similarly affecting the current patterns of population differentiation at the northern edge of their range in southern Québec. This is of relevance, as this region is located at the front range of many temperate species that are expected to shift their distribution toward northern latitude (Berteaux et al. 2010). Overall, the mechanisms and signature of range expansion are still poorly understood (Sexton et al. 2009). In this context, the integration of genetic and phenotypic data, both reflecting mechanisms operating at differing temporal and geographic scales, can help better understand the patterns of poleward distribution shifts of species and build scenarios for the future faunal turnover anticipated in response to global warming (Berteaux et al. 2010; Auzel et al. 2012).
